# Supplementary material for: The interactome of intact mitochondria by cross-linking mass spectrometry provides evidence for coexisting respiratory supercomplexes
Source: Mol Cell Proteomics. 2017 Dec 8;17(2):216–32. doi: 10.1074/mcp.RA117.000470 (PMC5795388; doi:10.1074/mcp.RA117.000470)
Supplement: Supplemental Data [file supp_RA117.000470_133922_0_supp_23978_fzffwf.zip › spectra_annotation/mito_DR_spectra_annotation/294-1-2-1-6-1.pdf]

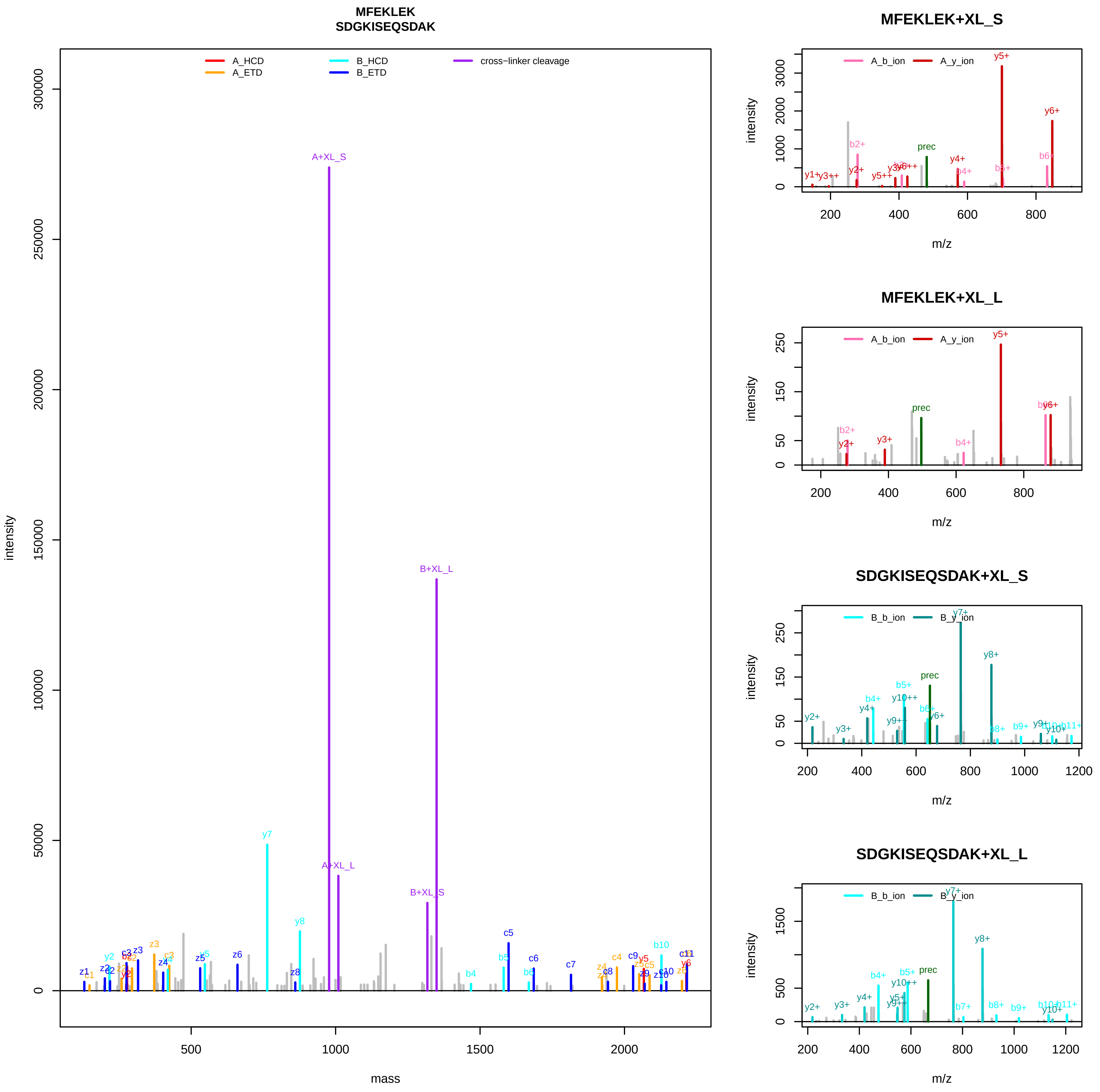

MFEKLEK
SDGKISEQSDAK
A_HCD
A_ETD
B_HCD
B_ETD
cross−linker cleavage
A+XL_S
B+XL_L
A+XL_L
B+XL_S
y7
y8
b10
c5
c11
intensity
mass
0
50000
100000
150000
200000
250000
300000
500
1000
1500
2000
MFEKLEK+XL_S
A_b_ion
A_y_ion
y5+
y6+
prec
y4+
b2+
b6+
m/z
MFEKLEK+XL_L
A_b_ion
A_y_ion
y5+
y6+
prec
y3+
b4+
b2+
y2+
m/z
SDGKISEQSDAK+XL_S
B_b_ion
B_y_ion
y7+
y8+
prec
b5+
y10++
b4+
y4+
y6+
y2+
y3+
m/z
SDGKISEQSDAK+XL_L
B_b_ion
B_y_ion
y7+
y8+
prec
b5+
y10++
b4+
y4+
y2+
y3+
b8+
b9+
b11+
m/z
